# Supplementary material for: Nature of Charge Transfer Effects in Complexes of Dopamine Derivatives Adsorbed on Graphene-Type Nanostructures
Source: Int J Mol Sci. 2024 Sep 29;25(19):10522. doi: 10.3390/ijms251910522 (PMC11477014; doi:10.3390/ijms251910522)
Supplement: Supplementary file 1 [file ijms-25-10522-s001.zip › ijms-3205101-supplementary.pdf]

Supplementary Materials: Nature of charge transfer effects in complexes of dopamine derivatives adsorbed on graphene-type nanostructures

Alex-Adrian Farcaş [1] and Attila Bende [1,*]

Contents

1. Electronic excited states for DsQ molecule. 1
1.1. Molecular orbitals 1
1.2. Natural difference orbitals 2

2. Electronic excited states for GrNP – DsQ binary complex. 2
2.1. Natural difference orbitals 2
2.2. Natural transition orbitals 5
2.3. Molecular orbitals 8

1. Electronic excited states for DsQ molecule.

1.1. Molecular orbitals

Table S1. The frontier (HOMO and LUMO) orbitals of the isolated DsQ computed at ωB97X-D3BJ/def2-TZVPP level of theory.

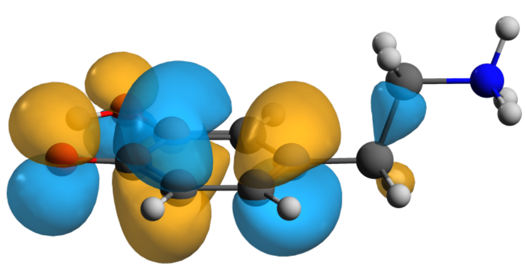	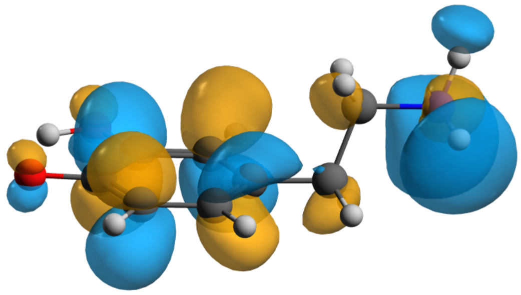
HOMO (H), $E = -6.92\,eV$	LUMO (L), $E = 2.47\,eV$
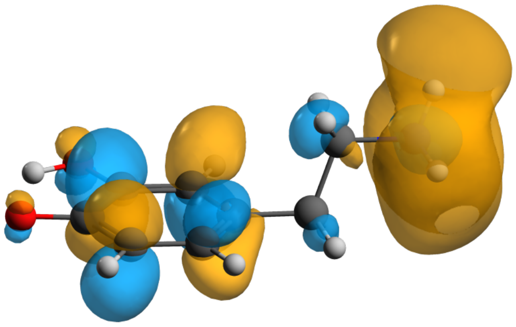	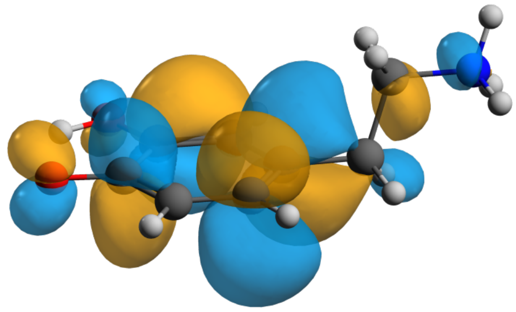
LUMO + 1 (L1)	LUMO + 2 (L2)

1.2. Natural difference orbitals

Table S2. The natural difference orbitals (NDO) between the ground and the given electronic excited state computed for the DsQ at TDDFT/ωB97X-D3BJ/def2-TZVPP level of theory (Green = hole, Yellow = electron density).

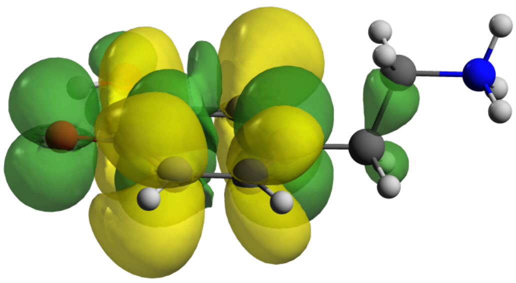	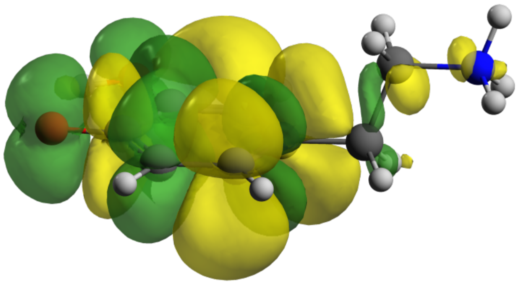
$S_0 \rightarrow S_1$ (259 nm)<br>H → L (53%), H → L + 1 (32%)	$S_0 \rightarrow S_2$ (224 nm)<br>H → L + 2 (78%)

2. Electronic excited states for GrNP – DsQ binary complex.

2.1. Natural difference orbitals

Table S3. The natural difference orbitals (NDO) between the ground and the given electronic excited state computed for the GrNP – DsQ binary complex at TDDFT/ωB97X-D3BJ/def2-TZVPP level of theory. (Green = hole, Yellow = electron density).

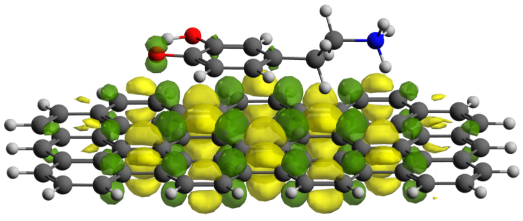	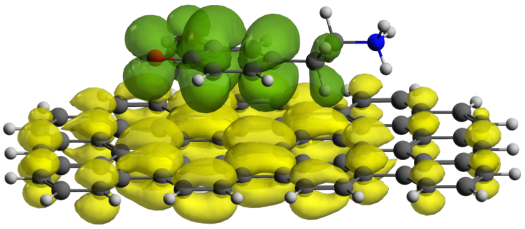
$S_0 \rightarrow S_1$ (570 nm)<br>H → L (92%)	$S_0 \rightarrow S_2$ (471 nm)<br>H - 1 → L (94%)
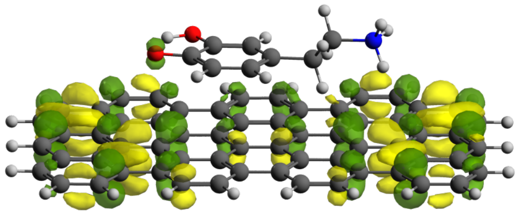	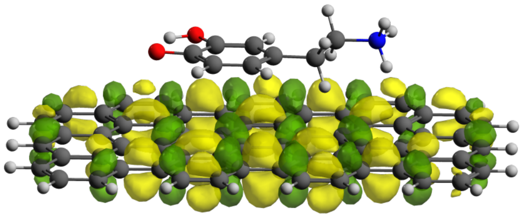
$S_0 \rightarrow S_3$ (384 nm)<br>H → L + 1 (50%)<br>H - 2 → L (38%)	$S_0 \rightarrow S_4$ (375 nm)<br>H → L + 2 (58%)<br>H - 3 → L (18%)
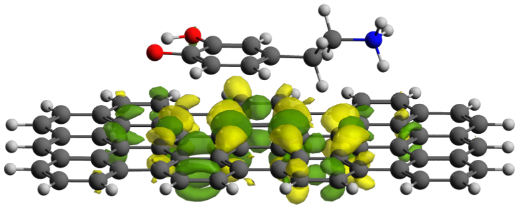	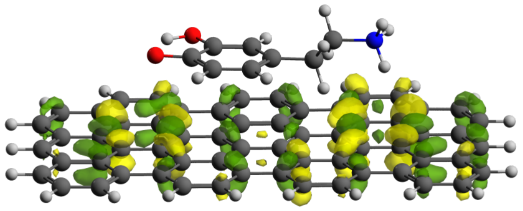
$S_0 \rightarrow S_5$ (364 nm)<br>H → L + 4 (26%), H - 4 → L (23%)<br>H → L + 3 (13%)	$S_0 \rightarrow S_6$ (357 nm)<br>H → L + 3 (31%), H - 3 → L (13%)<br>H - 6 → L (13%)

Table S3. (*cont.*) The natural difference orbitals (NDO) between the ground and the given electronic excited state computed for the GrNP – DsQ binary complex at TDDFT/ωB97X-D3BJ/def2-TZVPP level of theory. (Green = hole, Yellow = electron density).

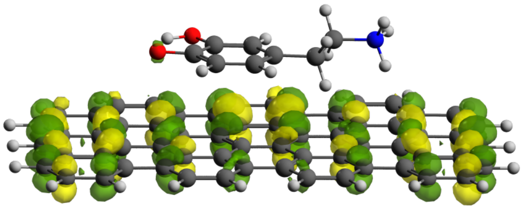	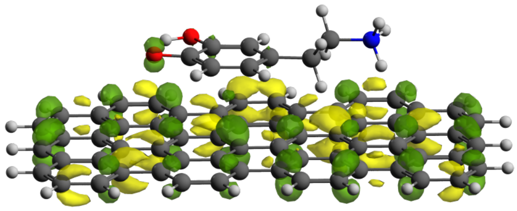
$S_0 \rightarrow S_7$ (340 nm)<br>H → L + 5 (38%), H → L + 4 (10%)	$S_0 \rightarrow S_8$ (325 nm)<br>H - 2 → L (39%), H → L + 1 (30%)
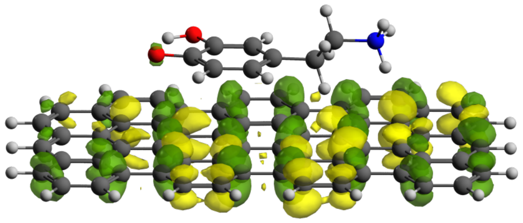	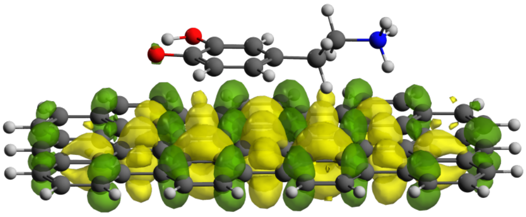
$S_0 \rightarrow S_9$ (322 nm)<br>H → L + 6 (15%), H → L + 2 (15%)<br>H - 3 → L (15%), H - 4 → L (12%)	$S_0 \rightarrow S_{10}$ (314 nm)<br>H → L + 7 (46%)<br>H → L + 6 (23%)
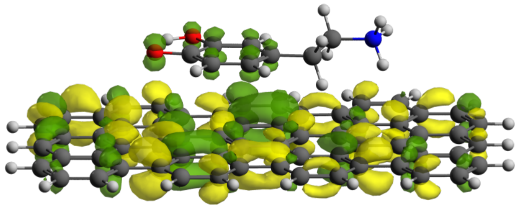	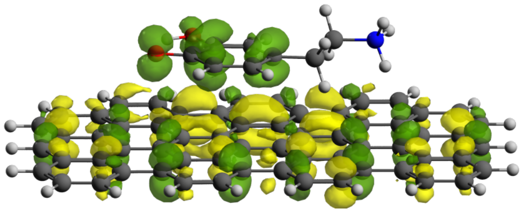
$S_0 \rightarrow S_{11}$ (305 nm)<br>H → L + 9 (16%)<br>H - 8 → L (12%)	$S_0 \rightarrow S_{12}$ (304 nm)<br>H → L + 7 (13%)<br>H → L + 2 (10%)
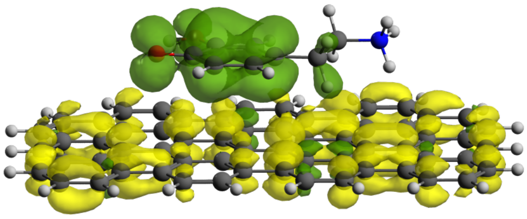	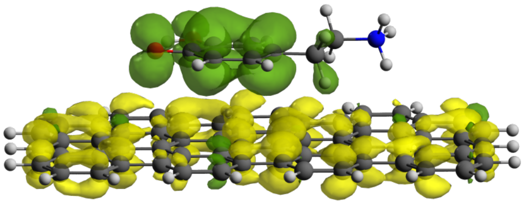
$S_0 \rightarrow S_{13}$ (301 nm)<br>H - 1 → L + 1 (43%)<br>H → L + 8 (13%), H - 2 → L + 1 (11%)	$S_0 \rightarrow S_{14}$ (297 nm)<br>H - 1 → L + 1 (27%)
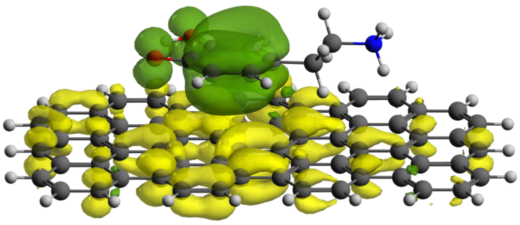	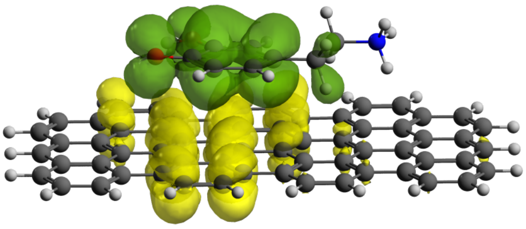
$S_0 \rightarrow S_{15}$ (295 nm)<br>H - 5 → L (33%)<br>H - 4 → L (10%)	$S_0 \rightarrow S_{16}$ (290 nm)<br>H - 1 → L + 4 (72%)

Table S3. (*cont.*) The natural difference orbitals (NDO) between the ground and the given electronic excited state computed for the GrNP – DsQ binary complex at TDDFT/ωB97X-D3BJ/def2-TZVPP level of theory. (Green = hole, Yellow = electron density).

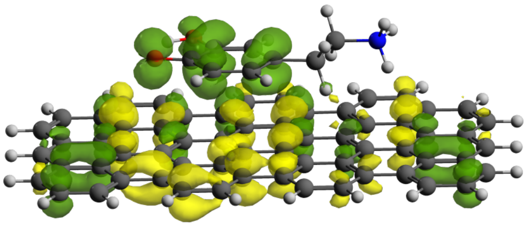	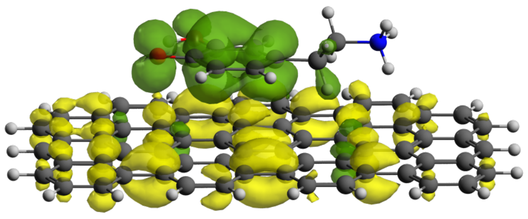
$S_0 \rightarrow S_{17}$ (285 nm)<br>H → L + 9 (15%)<br>H - 8 → L (15%), H → L + 5 (11%)	$S_0 \rightarrow S_{18}$ (284 nm)<br>H - 1 → L + 2 (33%)<br>H - 6 → L (21%)
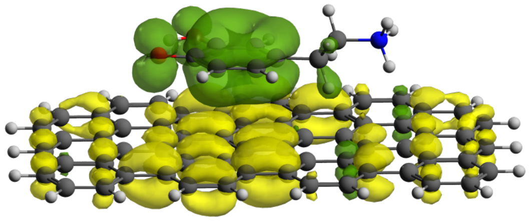	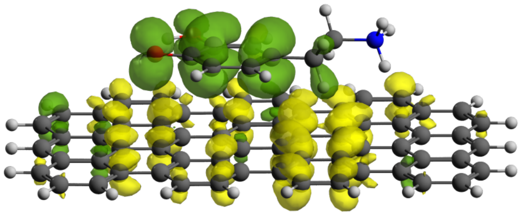
$S_0 \rightarrow S_{19}$ (282 nm)<br>H - 1 → L + 2 (33%)<br>H - 6 → L (27%)	$S_0 \rightarrow S_{20}$ (273 nm)<br>H - 1 → L + 3 (22%)<br>H - 1 → L + 5 (10%)
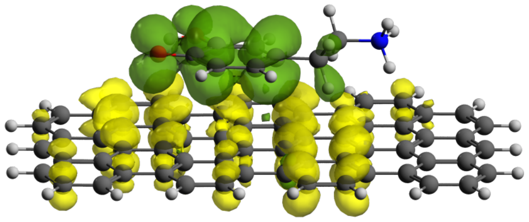	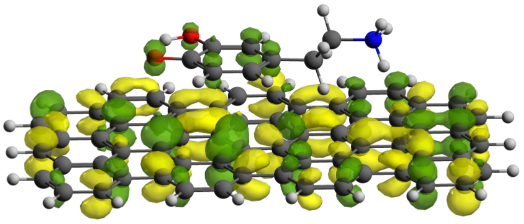
$S_0 \rightarrow S_{21}$ (270 nm)<br>H - 1 → L + 3 (28%)<br>H - 1 → L + 5 (21%)	$S_0 \rightarrow S_{22}$ (268 nm)<br>H → L + 8 (15%)<br>H - 9 → L (12%), H - 8 → L (10%)
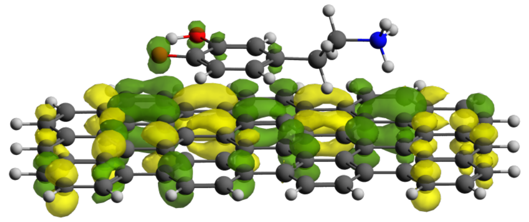	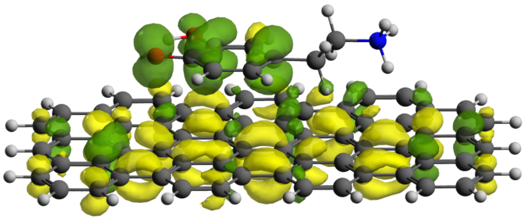
$S_0 \rightarrow S_{23}$ (266 nm)<br>H - 9 → L (12%)<br>H - 10 → L (10%)	$S_0 \rightarrow S_{24}$ (262 nm)<br>H - 12 → L (22%)
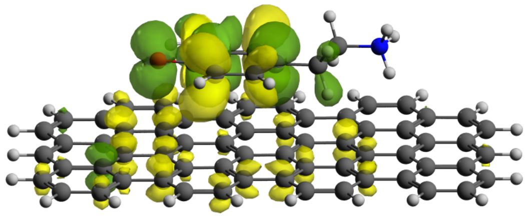	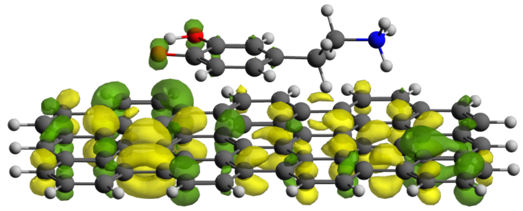
$S_0 \rightarrow S_{25}$ (261 nm)<br>H - 1 → L + 15 (20%)	$S_0 \rightarrow S_{26}$ (260 nm)<br>H - 12 → L (20%)<br>H → L + 11 (10%)

Table S3. (*cont.*) The natural difference orbitals (NDO) between the ground and the given electronic excited state computed for the GrNP – DsQ binary complex at TDDFT/ωB97X-D3BJ/def2-TZVPP level of theory. (Green = hole, Yellow = electron density).

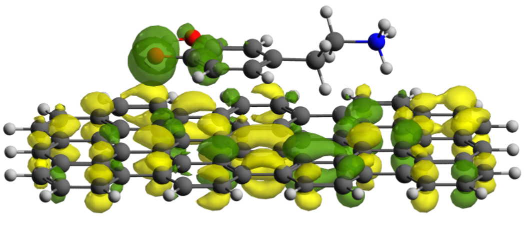	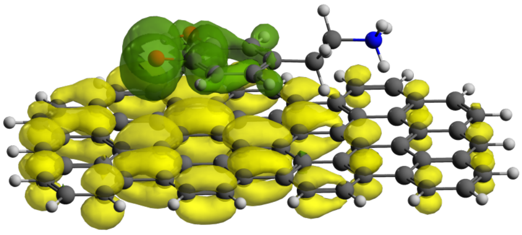
$S_0 \rightarrow S_{27}$ (259 nm)<br>H - 11 → L (24%), H - 7 → L (13%)	$S_0 \rightarrow S_{28}$ (258 nm)<br>H - 7 → L (57%), H - 8 → L (12%)
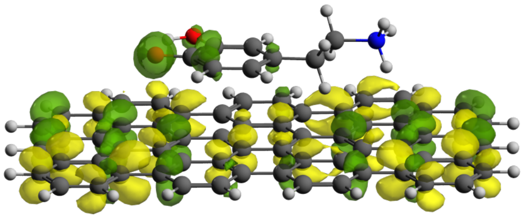	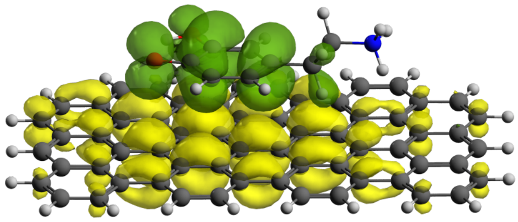
$S_0 \rightarrow S_{29}$ (256 nm)<br>H → L + 10 (23%)	$S_0 \rightarrow S_{30}$ (254 nm)<br>H - 1 → L + 7 (39%), H - 1 → L + 6 (20%)

2.2. Natural transition orbitals

Table S4. The natural transition orbitals (NTO) between the ground and the given electronic excited state computed for the GrNP – DsQ binary complex at TDDFT/ωB97X-D3BJ/def2-TZVPP level of theory. (Dark red = hole, Cian = electron density).

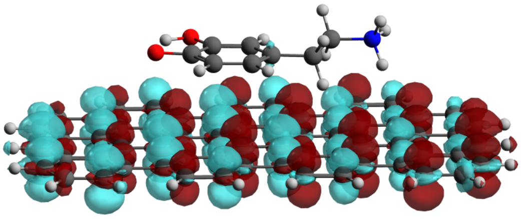	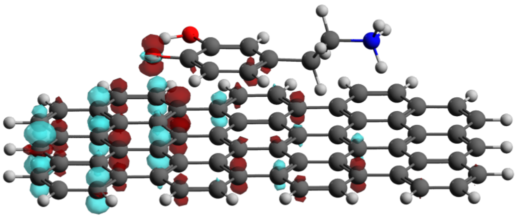
$S_0 \rightarrow S_1$ (570 nm)	$S_0 \rightarrow S_2$ (471 nm)
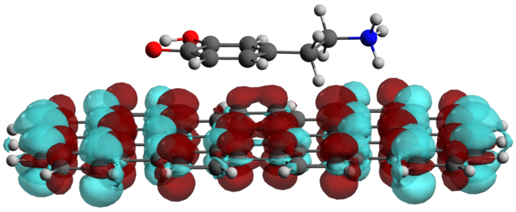	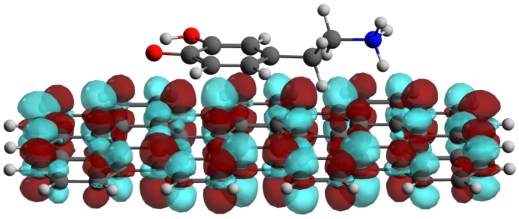
$S_0 \rightarrow S_3$ (384 nm)	$S_0 \rightarrow S_4$ (375 nm)
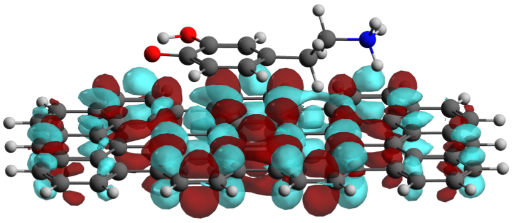	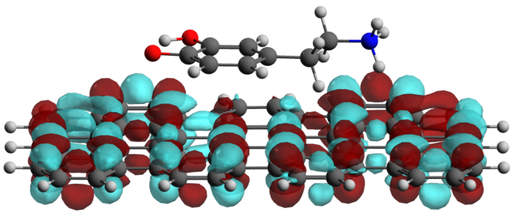
$S_0 \rightarrow S_5$ (364 nm)	$S_0 \rightarrow S_6$ (357 nm)

Table S4. (*cont.*) The natural transition orbitals (NTO) between the ground and the given electronic excited state computed for the GrNP – DsQ binary complex at TDDFT/ωB97X-D3BJ/def2-TZVPP level of theory. (Dark red = hole, Cian = electron density).

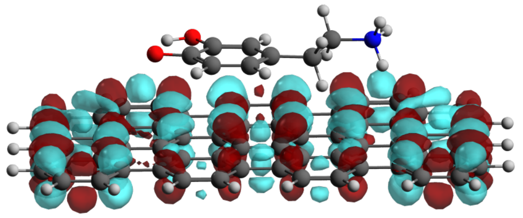	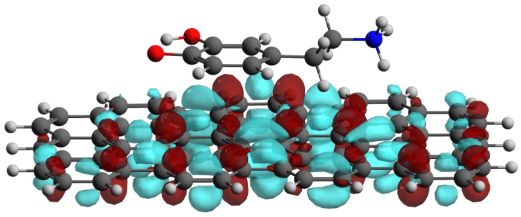
$S_0 \rightarrow S_7$ (340 nm)	$S_0 \rightarrow S_8$ (325 nm)
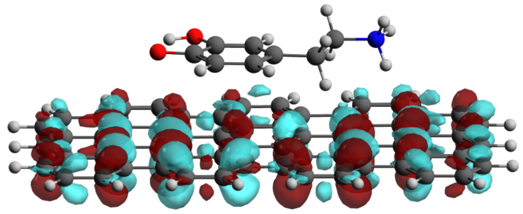	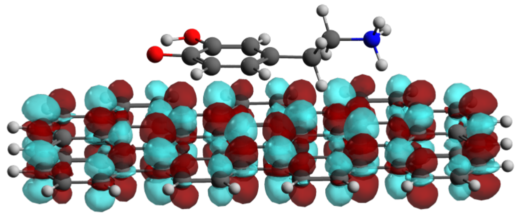
$S_0 \rightarrow S_9$ (322 nm)	$S_0 \rightarrow S_{10}$ (314 nm)
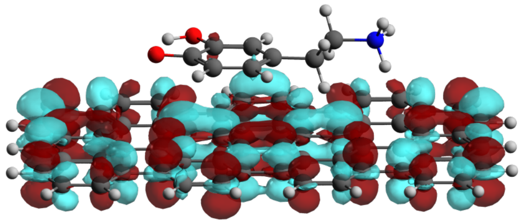	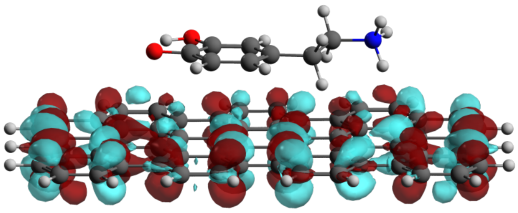
$S_0 \rightarrow S_{11}$ (305 nm)	$S_0 \rightarrow S_{12}$ (304 nm)
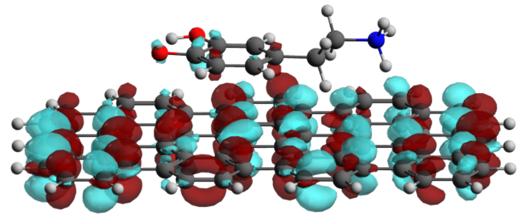	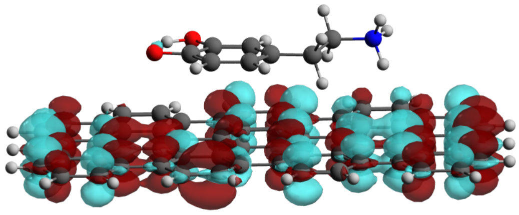
$S_0 \rightarrow S_{13}$ (301 nm)	$S_0 \rightarrow S_{14}$ (297 nm)
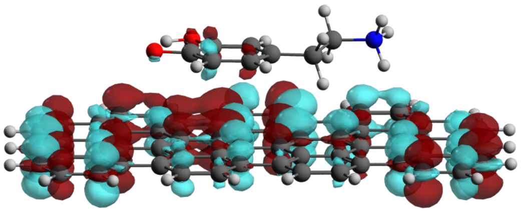	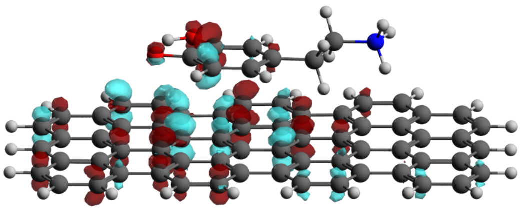
$S_0 \rightarrow S_{15}$ (295 nm)	$S_0 \rightarrow S_{16}$ (290 nm)
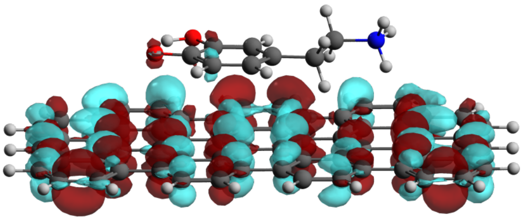	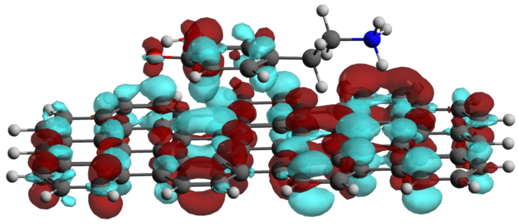
$S_0 \rightarrow S_{17}$ (285 nm)	$S_0 \rightarrow S_{18}$ (284 nm)

Table S4. (*cont.*) The natural transition orbitals (NTO) between the ground and the given electronic excited state computed for the GrNP – DsQ binary complex at TDDFT/ωB97X-D3BJ/def2-TZVPP level of theory. (Dark red = hole, Cian = electron density).

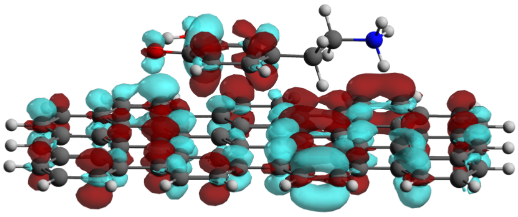	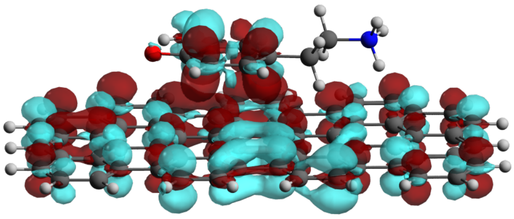
$S_0 \rightarrow S_{19}$ (282 nm)	$S_0 \rightarrow S_{20}$ (273 nm)
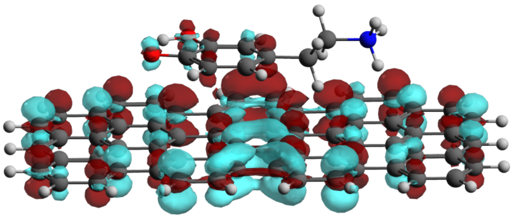	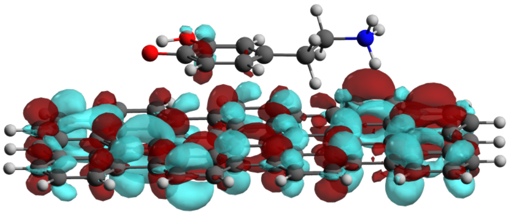
$S_0 \rightarrow S_{21}$ (270 nm)	$S_0 \rightarrow S_{22}$ (268 nm)
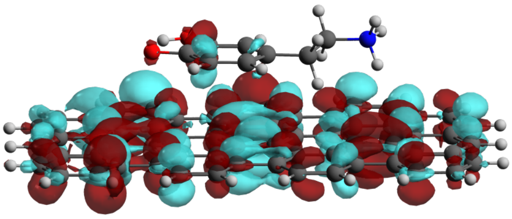	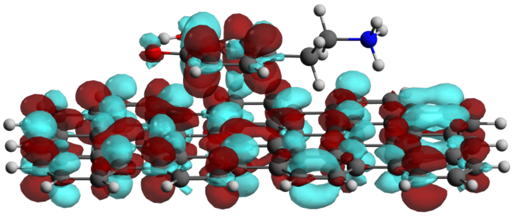
$S_0 \rightarrow S_{23}$ (266 nm)	$S_0 \rightarrow S_{24}$ (262 nm)
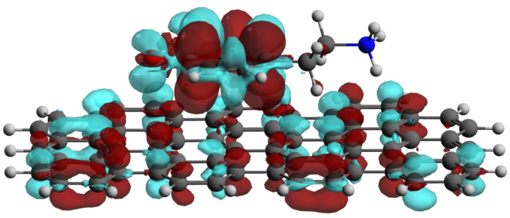	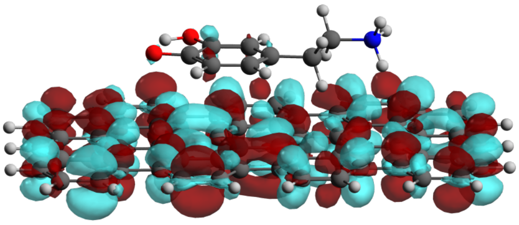
$S_0 \rightarrow S_{25}$ (261 nm)	$S_0 \rightarrow S_{26}$ (260 nm)
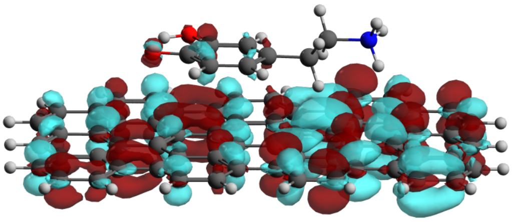	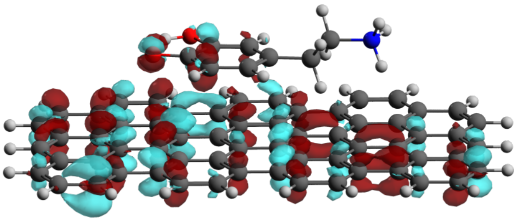
$S_0 \rightarrow S_{27}$ (259 nm)	$S_0 \rightarrow S_{28}$ (258 nm)
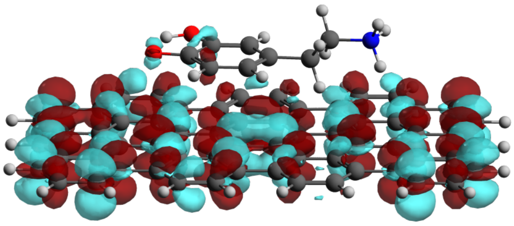	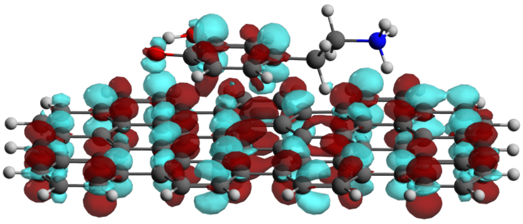
$S_0 \rightarrow S_{29}$ (256 nm)	$S_0 \rightarrow S_{30}$ (254 nm)

2.3. Molecular orbitals

Table S5. The occupied and unoccupied orbitals of the GrNP – DsQ binary complex computed at ωB97X-D3BJ/def2-TZVPP level of theory.

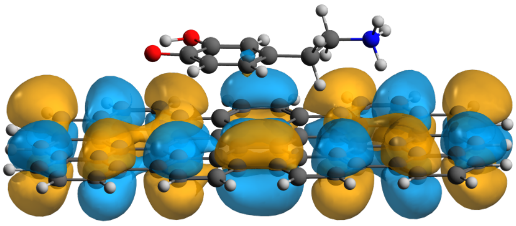	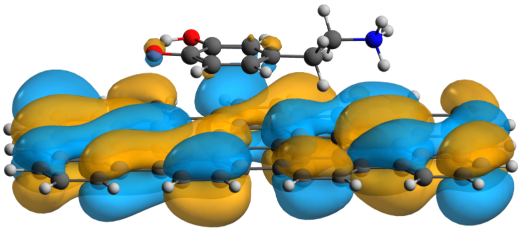
HOMO - 10	HOMO - 9
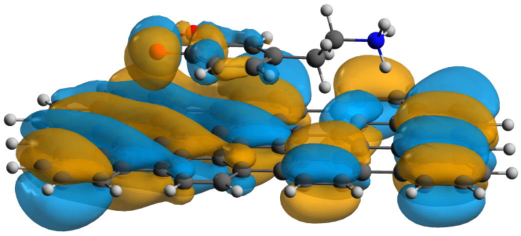	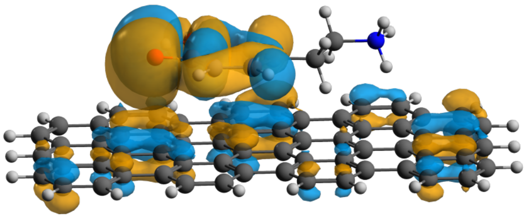
HOMO - 8	HOMO - 7
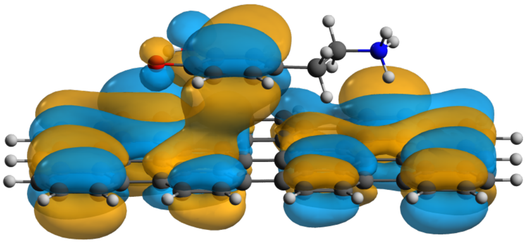	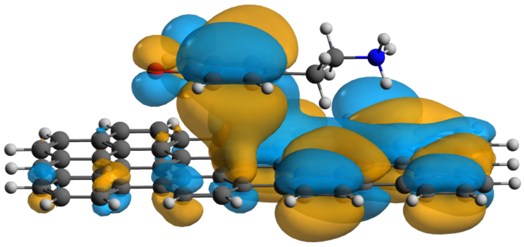
HOMO - 6	HOMO - 5
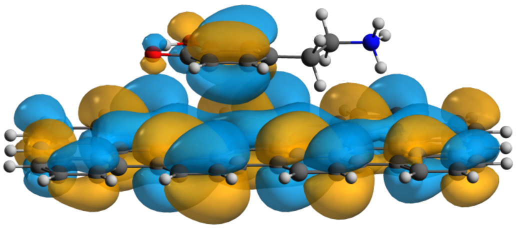	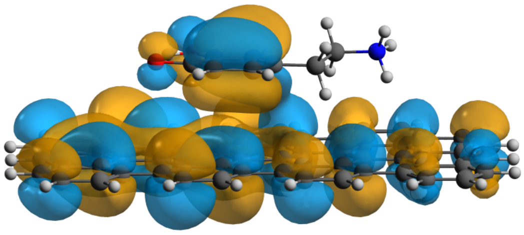
HOMO - 4	HOMO - 3
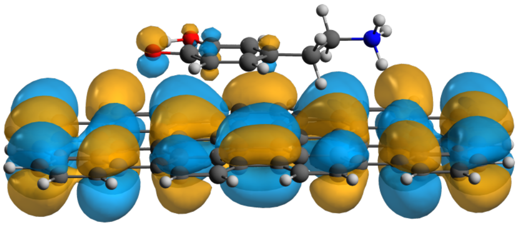	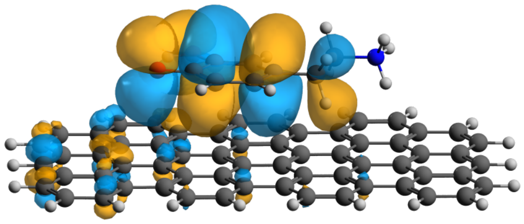
HOMO - 2	HOMO - 1

Table S5. (*cont.*) The occupied and unoccupied orbitals of the GrNP – DsQ binary complex computed at ωB97X-D3BJ/def2-TZVPP level of theory.

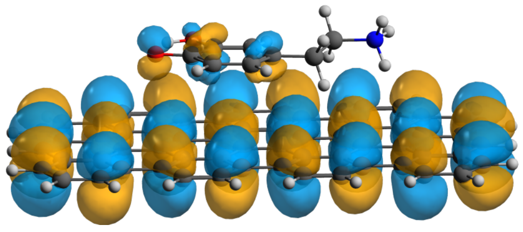	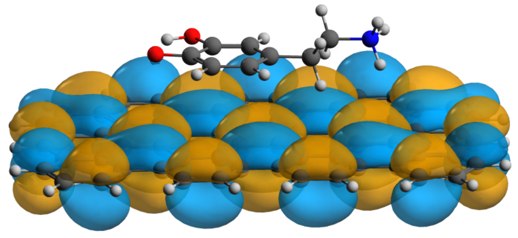
HOMO $E = -6.22\,eV$	LUMO $E = -1.58\,eV$
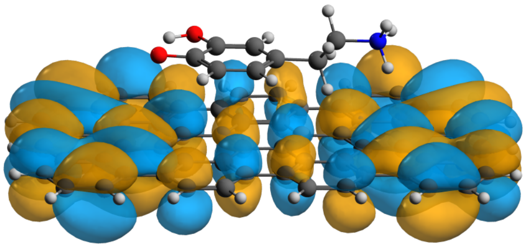	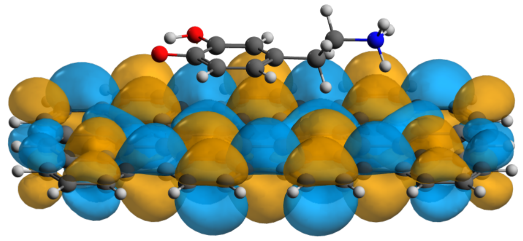
LUMO + 1	LUMO + 2
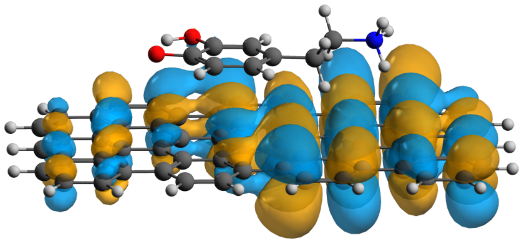	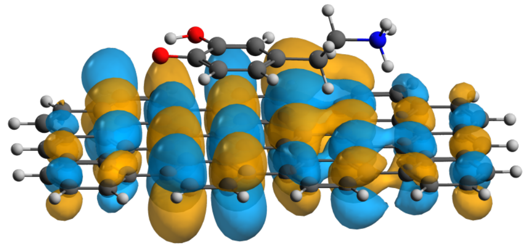
LUMO + 3	LUMO + 4
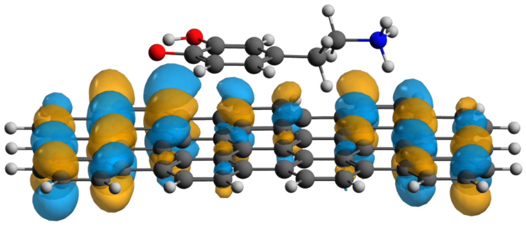	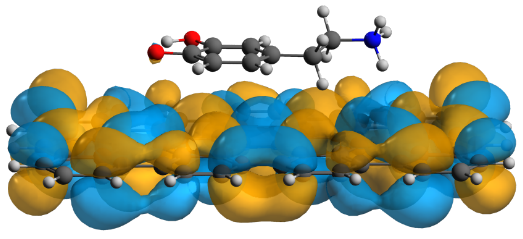
LUMO + 5	LUMO + 6
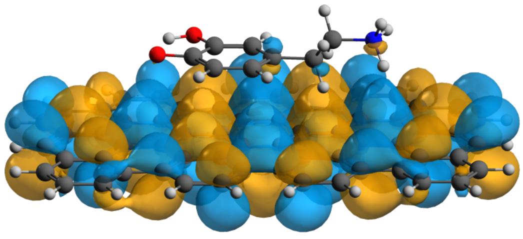	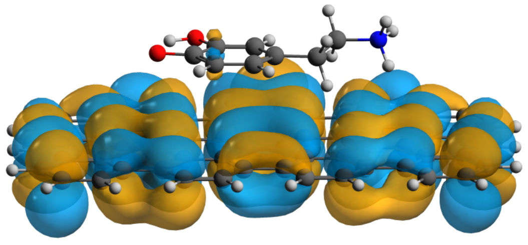
LUMO + 7	LUMO + 8

Table S5. (*cont.*) The occupied and unoccupied orbitals of the GrNP – DsQ binary complex computed at ωB97X-D3BJ/def2-TZVPP level of theory.

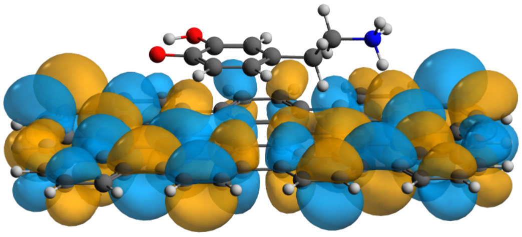	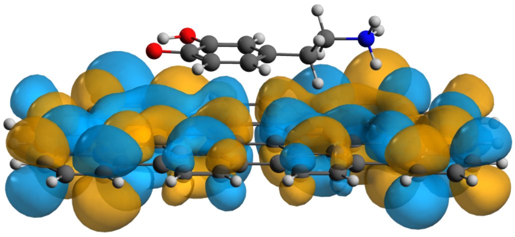
LUMO + 9	LUMO + 10
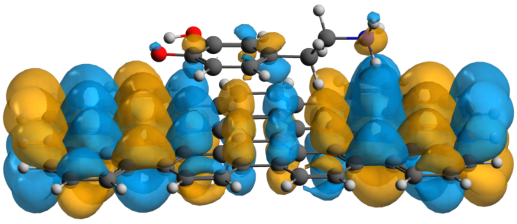	
LUMO + 11	
